# A high trans-zeatin nucleoside concentration in corms may promote the multileaf growth of *Amorphophallus muelleri*

**DOI:** 10.3389/fpls.2022.964003

**Published:** 2022-10-06

**Authors:** Zhiqin Xue, Feiyan Huang, Jiani Liu, Yanguo Ke, Huanyu Wei, Penghua Gao, Ying Qi, Lei Yu

**Affiliations:** Yunnan Urban Agricultural Engineering and Technological Research Center, College of Agronomy, Kunming University, Kunming, China

**Keywords:** hormones, trans-zeatin nucleoside, cytokinins, multileaf growth, konjac

## Abstract

*Amorphophallus muelleri* has a multileaf growth pattern different from that of other konjacs; however, the hormonal mechanism underlying this phenomenon is not clear. In this study, the levels of hormones closely related to the sprouting of the axillary bud, including five types of cytokinins, indole-3-acetic acid (IAA) and abscisic acid (ABA) were measured. In the second leaf sprouting stage, the content of trans-zeatin riboside (tZR) in corms increased more than 5000-fold over that in the dormancy period. Surprisingly, although the expression of *CYP735A1* and *CYP735A2*, which synthesize the precursors for tZR was elevated at the second leaf sprouting stage, the expression of *IPTs*, which have key roles in cytokinin biosynthesis, did not change significantly. In addition, most cytokinin contents in leaves during the same period were significantly lower than those in corms. We speculate that the high cytokinin contents in the corms may not biosynthesized *de novo* in corms. In addition, the IAA content in the corms also considerably increased during the second leaf sprouting stage. Indole-3-acetaldehyde oxidase (*AO1*) and auxin efflux carrier *PIN1A*, presented relatively high expression levels in the same period. In contrast, ABA content, and the expression of *NCED1*, a rate-limiting enzyme in ABA biosynthesis, were suppressed at the second leaf sprouting stage. It is worth mentioning that N6-(Δ2-isopentenyl) adenosine (iP)-type cytokinins have a high content in corms in the dormant period that significantly decreases after the first leaf sprouting stage, which is completely different from the trend of tZR. By treating dormant corms with iP, the percentage of multibud plants increased, and the growth performance in terms of bud and root length was significantly higher than those of the control. This implies that iP-type cytokinins tend to play a role in promoting first seedling sprouting. Furthermore, there was a remarkable increase of the IAA content in both corms and roots under iP treatment but an inhibitory effect in buds. We speculate that the increase in the IAA content induced by iP is tissue specific. These results will assist in the understanding of the role of hormones, especially cytokinins, in the multileaf growth type of konjac.

## 1 Introduction

**Figure 1:**
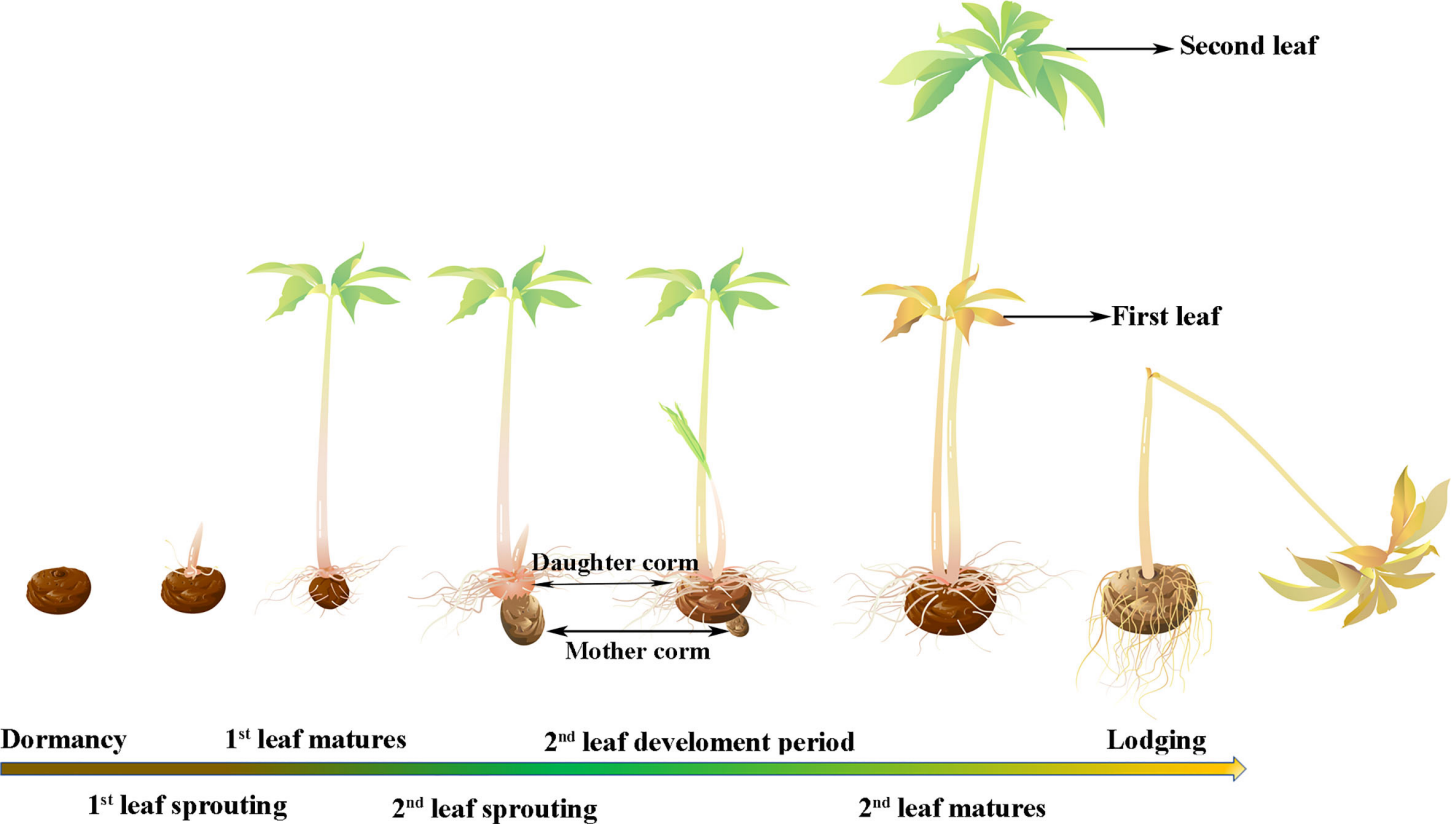
Schematic representation of the multileaf growth pattern of *A. muelleri*.

Konjac is a common name for a genus of *Amorphophallus* in the family Araceae. *Amorphophallus* plants are perennial geophytes that usually consist of an underground corm, which is the main source of glucomannan (Zhang and Wang, 2010; Pouchon et al., 2022). Konjac glucomannan (KGM) is a natural polymer polysaccharide with strong water absorption, a high swelling rate and a low caloric value, so it has various beneficial pharmacological effects, including weight loss, antidiabetic and antioxidant properties (Devaraj et al., 2018). Because of its high content of glucomannan and other nutritional value, konjac has become one of the most popular cash crops in Southwest China in recent years. The pearl-bud type of konjac refers to a group of konjacs that can produce pearl-shaped aerial reproductive corms on the leaf surface (at the intersection of leaf veins). This type of konjac is represented by *Amorphophallus muelleri*, which has the same biogeographic distribution as *A*. *konjac* (the current main cultivar in Asia); however, *A. muelleri* and *A. konjac* belong to different clades on phylogenetic trees, the Continental Asia I and Continental Asia II clades, respectively (Pouchon et al., 2022). *A. muelleri* has the characteristic of multiple leaves growing sequentially that together feed one corm. This is significantly different from *A. konjac*, which bears a single leaf per growing season. With the first leaf fully expanded, the mother corm gradually shrivels and is replaced by the daughter corm, after which the lateral buds sprout, growing into a stronger second leaf with a larger leaf area, and all the leaves work together to maintain the growth of the new corm (**Figure 1**). This multileaf growth pattern did not reduce the glucomannan content of pearl-bud-type of konjac; instead, the glucomannan content reached 60-75%, and its viscosity and transparency were also significantly better than those of *A. konjac* (Zhang and Wang, 2010). Therefore, the multileaf growth pattern improves the expansion coefficient of the corm, shortens the planting time, and has high economic benefits. However, the mechanism of how pearl-bud-type konjacs achieve sequential growth of multiple leaves is not clear.

The second leaf of *A. muelleri* sprouts from the axils of the first seedling, and the sprouting of the axillary bud is closely related to nitrogen and light (Schneider et al., 2019; Wang R. et al., 2020), but other factors, such as the hormonal levels in different tissues, are also important. Endogenous plant hormones and their relative balance are suggested to regulate endodormancy and sprouting (Suttle, 2009; Sorce et al., 2009; Hartmann et al., 2011). Abscisic acid (ABA) has been associated with the maintenance of corm dormancy (Suttle and Hultstrand, 1994), and decreasing the ABA content or inhibiting its synthetic genes can induce dormancy release and sprouting (Wang Z. et al., 2020; Tosetti et al., 2021).

Cytokinins are involved in various aspects of developmental processes, including senescence, apical dominance, root proliferation, phyllotaxis, and reproductive competence (Sakakibara, 2006). Cytokinins that are directly applied to buds can promote bud outgrowth (Domagalska and Leyser, 2011). In contrast, auxin has long been considered to play a central role in the inhibition of axillary bud outgrowth. This has been demonstrated in several species through auxin precursor­deficient mutants (Beveridge et al., 1997a; Sorefan et al., 2003; Arite et al., 2007). As the most important natural auxin in plants, indole-3-acetic acid (IAA) is mainly synthesized from the amino acid tryptophan (Zhao, 2014). First, tryptophan is converted into indole-3-pyruvate by tryptophan aminotransferase (TAA, Stepanova et al., 2008) and then converted into IAA catalyzed by YUCCA of the flavin monooxygenase family. IAA has also been observed to regulate cytokinin production; for example, a decrease in the level of IAA leads to strong cytokinin synthesis by regulating the expression of members of the adenylate isopentenyltransferase (IPT) family, which have vital roles in cytokinin biosynthesis (Tanaka et al., 2006; Domagalska and Leyser, 2011). Cytokinins can reduce the output of IAA through the Aux/IAA protein SHY2 and redistribute IAA. IAA in turn promotes SHY2 degradation to maintain the level of IAA (Schaller et al., 2015).

The pathways involved in the biosynthesis of cytokinins have been well described (Zhao, 2008; Kudo et al., 2010), and it has been shown that localized cytokinin biosynthesis is also crucial for organ growth and patterning (Zhao, 2008). The first key step in cytokinin biosynthesis is to transfer an isopentenyl chain provided by dimethylallyl diphosphate to an adenine nucleotide (ATP, ADP, or AMP) catalyzed by IPTs (Kakimoto, 2001). These reaction products, such as isopentenyl adenosine-5’-triphosphate (IPTP) and isopentenyl adenosine-5’-diphosphate (IPDP), are subsequently hydroxylated to zeatin monophosphate (ZMP) by cytochrome P450 monooxygenase (CYP735A). The last step is the removal of the ribose group catalyzed by LONELY GUY (LOG; Kurakawa et al., 2007). The degradation of cytokinins is primarily catalyzed by cytokinin oxidase/dehydrogenase (CKX, Kudo et al., 2010; Wu et al., 2017).

N6-(Δ2-isopentenyl) adenosine (iP) and trans-zeatin (tZ) are considered biologically active compounds due to their higher relative abundance and affinity for their receptors (Osugi and Sakakibara, 2015). In some plants, including major crops (for example, maize and rice), iP and tZ assume a central role in cytokinin activities (Lomin et al., 2011; Kamada-Nobusada et al., 2013). Trans-zeatin riboside (tZR) is a riboside of trans-zeatin that is used for the direct initiation of shoot cultures and for increasing the sprouting of lateral buds (Kuroha et al., 2002). In contrast, tZR has shown significant inhibitory effects on adventitious root formation (Beveridge et al., 1997b). These results suggest that tZR-type cytokinins may have different effects in different tissues, which also indicates that it is necessary to determine the concentration of cytokinins in different organs when exploring the effect of cytokinin on plant growth.

Since plant hormones are closely related to dormancy or germination, levels of hormones show dynamic changes during plant development, and exploring the hormone levels and the relationships between the hormones in individual tissues is crucial. Cytokinins and auxin are largely specific to axillary meristems and buds, and their interactions are critical to organ formation, which makes them good candidates for a regulatory role in bud activation. Although several studies have discussed the effects of hormones on plant development, few studies have addressed the hormonal patterns that trigger multiple leaf growth in pearl-bud type konjac. In this paper, we mainly focused on cytokinins and discussed the interaction between cytokinins and IAA and ABA during axillary bud growth of pearl-bud-type konjacs.

## 2 Materials and methods

### 2.1 Plant growth conditions and sample collection

Two-year-old corms of *A. muelleri*, which weighed approximately 100 g and had a uniform size, were selected and grown in a greenhouse from May to October 2021 after being transported into 10 L plastic pots containing 8 kg substrate (white sod peat: white peat: peat fibers= 6:3:1, pH value: 6.0, NPK fertilizer level: 1.0 g L^-1^, Klasmann, Germany) at the Yunnan Urban Agricultural Engineering and Technological Research Center, Kunming University (24.98°N, 102.79°E, altitude: 1934 m). The cultivation temperature was 25-35°C, under 50% shade conditions, with a relative humidity of 60-80%, and watering and fertilization were performed as needed.

As shown in **Figure 1**, corms that were verified to be dormant, and that had not sprouted after 2 weeks at 20°C (Campbell et al., 2008) were selected as materials for the dormancy period. The corms with buds that had germinated 3-4 cm were used as materials for the sprouting stage. The first fully expanded leaf was used as the maturation stage (approximately 21 days after sprouting). Sampling of materials in the second leaf sprouting stage was performed when the buds of the second seedling were 3-4 cm long (approximately 60 days after sprouting). The second leaf development period was considered when the bud was approximately 10 cm long and the leaves were clustered and unexpanded (approximately 75 days after sprouting). Similar to the first leaf, the full spread of the second leaf was considered to be mature (approximately 85 days after sprouting). When the second leaves withered and wilted, the plant was considered to be in the lodging period (more than 120 days after sprouting).

### 2.2 Measurements of endogenous phytohormones

#### 2.2.1 Sample extraction

Leaf samples were collected from the first leaf matures stage, the second leaf sprouting stage (because the second leaf had just emerged, the first leaves were selected as materials for this period), the second leaf development stage (from this stage, the samples were all taken from the second leaves), the second leaf matures stage and the lodging stage. Corm samples were taken at all stages. During first leaf maturity and second leaf emergence, the mother corm gradually shrinks and the daughter corm starts to develop (**Figure 1**), so the mother corms were selected at the first leaf maturity stage, and the daughter corms were sampled from the second leaf sprouting period to the later periods. Samples from three individual plants were mixed as one biological replicate and three biological replicates were used in the study.

Sample extraction was carried out following a high-throughput target detection method by Shanghai Biotree Biotechnology Co., Ltd. (Shanghai, China). In brief, samples were first ground in liquid nitrogen, and then 1000 μL of extract solution (50% acetonitrile in water, precooled at -40°C, containing isotopically labeled internal standard mixture) was added. Information about the isotopically labeled internal standard used in this study is shown in **Supplemental Data 1**. After vortexing for 30 seconds and sonicating for 5 minutes in an ice bath, the samples were homogenized at 40 Hz for 4 minutes. Following centrifugation at 12000 rpm for 15 min at 4°C, 90 μL of 10% ACN/H_2_O (v/v) was added. The samples were centrifuged once again, filtered through a 0.22 mm polytetrafluoroethylene membrane filter, and then subjected to ultra-high-performance liquid chromatography tandem mass spectrometry (UHPLC-MS/MS) analysis.

#### 2.2.2 UHPLC-MRM-MS analysis

A Waters ACQUITY UPLC CSH C18 column (150 * 2.1 mm, 1.7 mm, Waters Corporation, MA, USA) was used for UHPLC separation. Mobile phase A was 0.01% formic acid in water, and mobile phase B was 0.01% formic acid in acetonitrile. The column temperature was set at 50°C. The autosampler temperature was set at 4°C. The injection volume was 5 μL. The typical ion source parameters were as follows: curtain gas = 40 psi, ion spray voltage = ± 4500 V, temperature = 475°C, ion source gas 1 = 30 psi, and ion source gas 2 = 30 psi.

Flow injection analysis was used to optimize the multiple reaction monitoring (MRM) parameters for each targeted analyte, which was injected into the API source of the mass spectrum using the standard solutions of each analyte. MRM scan mode was used to optimize collision energy for each Q1/Q3 pair using some of the most sensitive transitions. The Q1/Q3 pairs that showed the best sensitivity and selectivity were selected as ‘quantifiers’ for quantitative monitoring among the optimized MRM transitions per analyte. An additional transition served as a ‘qualifier’ for identifying the target analytes. SCIEX Analyst Work Station Software (Version 1.6.3) and Sciex MultiQuant™ 3.0.3 were used for MRM data acquisition and processing. The extracted ion chromatographs (EICs) from a standard solution and a sample of the targeted analytes under the optimal conditions are shown in **Supplemental Figure 1**.

#### 2.2.3 Calibration curves

The calibration solutions were analyzed with UPLC-MRM-MS/MS using the methods described above. The y-axis represents the ratio of peak areas for analyte/IS, and the x- axis represents the concentration (nmol/L) for an analyte. The least-squares method was used for the regression fitting. For the curve fitting, 1/x weighting provided the highest accuracy and correlation coefficient (R^2^). Levels were excluded from calibration if their accuracy fell outside of 80%-120%.

#### 2.2.4 Precision and accuracy

The precision of the quantitation was measured by the relative standard deviation (RSD), determined by injecting analytical replicates of a QC sample. The accuracy of quantitation was measured by the analytical recovery of the QC sample. The percent recovery was calculated as [(mean observed concentration)/(spiked concentration)] × 100%.

### 2.3 RNA-seq analysis

The samples for RNA-seq analysis were taken at the corm (approximately 5 mm below the buds) at the first leaf maturity period, the second leaf sprouting period, the second leaf development period and the second leaf maturity period. Samples from three individual plants were mixed as one biological replicate, and three biological replicates were used in the study.

Total RNA was extracted using a TRIzol reagent kit (Invitrogen, Carlsbad, CA, USA) according to the manufacturer’s protocol. RNA quality was assessed on an Agilent 2100 Bioanalyzer (Agilent Technologies, Palo Alto, CA, USA) and checked using RNase free agarose gel electrophoresis. The Concentration, quality and integrity number (RIN value) of the RNA are shown in **Supplemental Data 2** and **Supplemental Figure 2**. 3 μg of RNA was used for RNA-Seq library construction. Sequencing was performed using an Illumina Novaseq6000 platform, and library construction as well as RNA-seq analysis were performed by Gene Denovo Biotechnology Co. (Guangzhou, China). The library fragments were purified with a QiaQuick PCR extraction kit (Qiagen, Venlo, The Netherlands) and ligated to Illumina sequencing adapters. To obtain high quality clean reads, reads were further filtered by fastp (Chen et al., 2018). The short reads alignment tool Bowtie2 (Langmead and Salzberg, 2012) was used for mapping reads to the RNA database. An index of the konjac reference genome (https://www.ncbi.nlm.nih.gov/sra/PRJNA608095) was built, and paired-end clean reads were mapped to the reference genome using HISAT2 (Kim et al., 2015) with “-rna-strandness RF” and other parameters set as a default. The mapped reads of each sample were assembled by using StringTie v1.3.1 (Pertea et al., 2015) in a reference-based approach. For each transcription region, a FPKM (fragment per kilobase of transcript per million mapped reads) value was calculated to quantify its expression abundance and variations, using RSEM software (Li and Dewey, 2011). Significant differential expression was determined using with an adjusted P value < 0.05 and fold change>2. The RNA-seq data used in the study have been deposited in the Gene Expression Omnibus (http://www.ncbi.nlm.nih.gov/bioproject/863734) under code PRJNA863734.

### 2.4 Exogenous applications of iP and growth measurements

To study the effects of iP on bud outgrowth, foliar corms in the dormant stage with uniform size and weighing approximately 5 g were selected and then immersed in 50 mg L^-1^ and 100 mg L^-1^ iP (Solarbio, Beijing, China) solution for 3 h (Janečková et al., 2019). The control was immersed in water under the same conditions. Then, the corms were planted into cultivation boxes with substrate (same as above) and transferred to an incubator with a relative humidity of 60% and light/dark photoperiod of 16/8 h at 28°C. The growth traits, such as bud length, were measured 10 days after bud sprouting (approximately 30 days after planting). Both the number of roots and buds were counted only for those with a length greater than 1 cm. The root length is the average of the three longest roots of each plant. After the measurements of growth traits, the buds, corms and roots of each plant from different treatments were collected and immediately placed in liquid nitrogen. Three biological replicates, each of which was a mixed sample from at least ten plants, were used in measurements of endogenous phytohormones.

### 2.5 Statistical analysis and graphing

For comparison of the changes in hormone content at different growth stages and the physiological traits among different treatments, one-way ANOVA was used followed by a post-hoc Tukey’s test using SPSS version 16.0 (SPSS Inc., USA), and P ≤ 0.05 was considered significant. Heatmaps were generated using TBtools (Chen et al., 2020), and other line plots were generated using SigmaPlot version 10.0 (Systat Software Inc., USA).

## 3 Results

### 3.1 Changes in cytokinin levels during the growth cycle of *A. muelleri*

**Figure 2:**
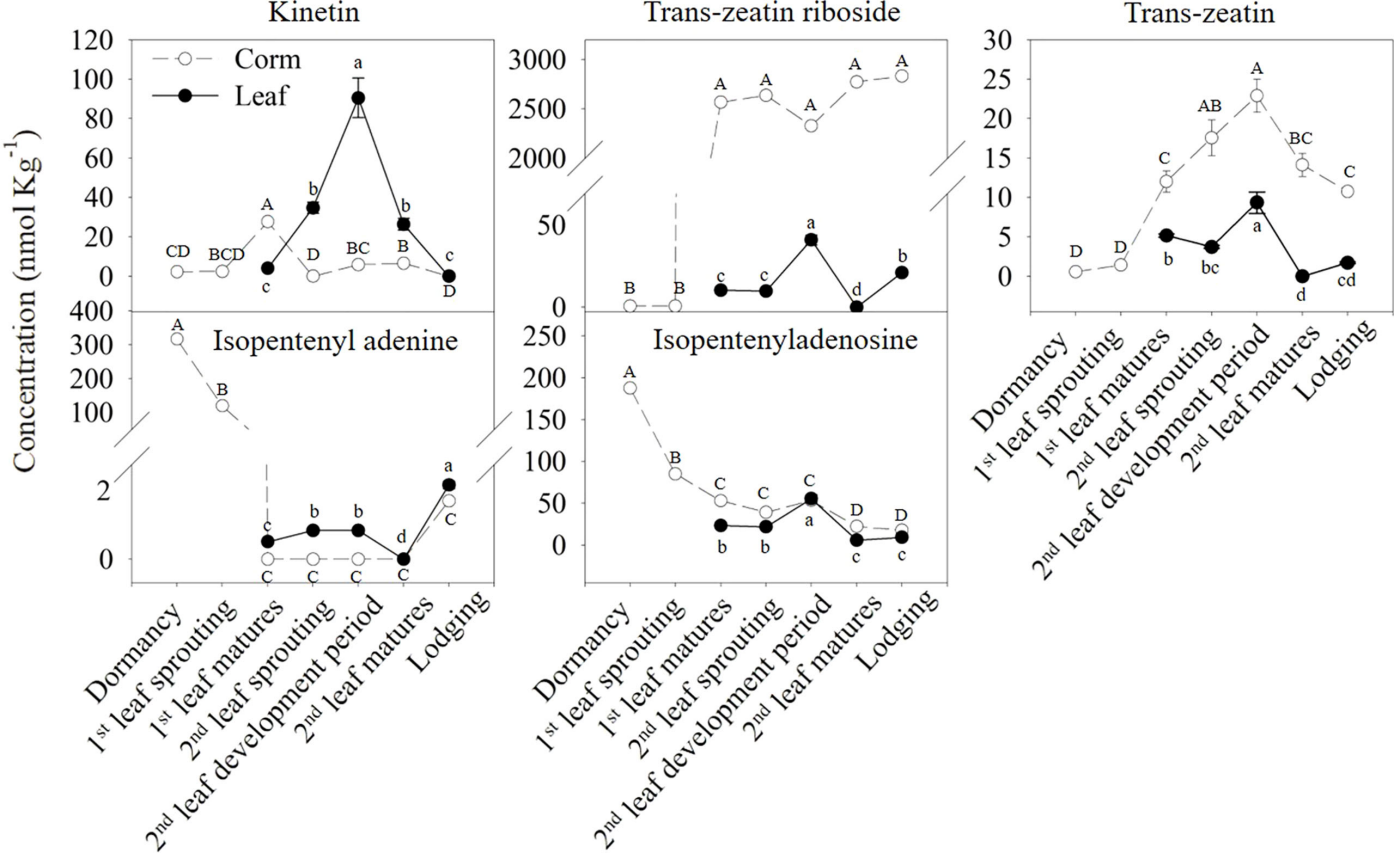
Differences in the concentrations of various types of cytokinins in the corms and leaves during the growth cycle of *A. muelleri*. The dashed lines with white dots represent corms; solid lines with black dots represent leaves. Error bars represent the SE (n=3), and different letters indicate significant differences compared with different growth periods (P < 0.05, based on ANOVA, followed by Tukey’s post-hoc tests for comparison).

To investigate the changes in cytokinins in corms and leaves at different growth stages, the contents of five types of cytokinins, including kinetin (KT), tZR, tZ, iP and isopentenyladenosine (iPA), were measured at different growth stages (**Figure 2**). KT, tZR, and tZ in corms showed an increasing trend during the first leaf emergence and maturation stages. Among these, the KT level peaked at the maturation of the first leaf, and the increasing trend of tZ was maintained until the growth period of the second leaf and started to decrease until maturation of the second leaf occurred. In contrast, iP and iPA in the corms showed a completely different trend, with the highest levels in the dormant period and significantly decreased with bud emergence, and this trend was not reversed until the lodging stage.

In particular, the tZR content in the corm showed the most drastic changes among all types of cytokinins, with a peak value of 2637.18 nmol kg^-1^ at the second leaf sprouting stage, which was more than 5000 times that of the dormant period. Compared to corms, the variation and content of most types of cytokinins in leaves were smaller and relatively low. The highest amount of KT in the leaves was found during the development of the second leaf, with a value of 90.47 nmol kg^-1^. It was also the most abundant cytokinin type in leaves.

### 3.2 Effect of external application of iP on growth traits and the iP, tZR, IAA and ABA contents of different tissues

To investigate the reason for the high iP-type cytokinin content in dormant corms, we treated foliar corms in the dormant period with different concentrations of iP solution. In terms of growth traits, 100 mg L^-1^ iP treatment significantly improved the length of buds and roots; the percentage of multibud plants (plants with two or more buds greater than 1 cm in length) and the number of roots also showed an increasing trend (**Table 1**). All growth traits data was shown in **Supplemental Data 3**.

**Table 1:** Effect of iP application on the growth traits of *A. muelleri*.

Traits	Treatment		
	Control	iP (50 mg L^-1^)	iP (100 mg L^-1^)
Fresh weight (g)	8.82 ± 0.36	7.56 ± 0.31*	8.31 ± 0.35
Bud length (cm)	4.05 ± 0.42	4.15 ± 0.40	5.75 ± 0.46*
Percentage of individuals with two or more buds (%)	3.23	6.45	18.75
Root length (cm)	6.85 ± 0.49	6.13 ± 0.59	9.43 ± 0.89*
Number of roots	5.71 ± 0.58	5.39 ± 0.44	7.06 ± 0.42

Data are expressed as the mean ± SE (n ≥ 10). Significant differences (compared to control; based on one way ANOVA, followed by Tukey’s post-hoc tests for comparison) at P<0.05 are indicated with *. All growth traits data used to build this table is shown in **Supplemental Data 3**.

**Figure 3:**
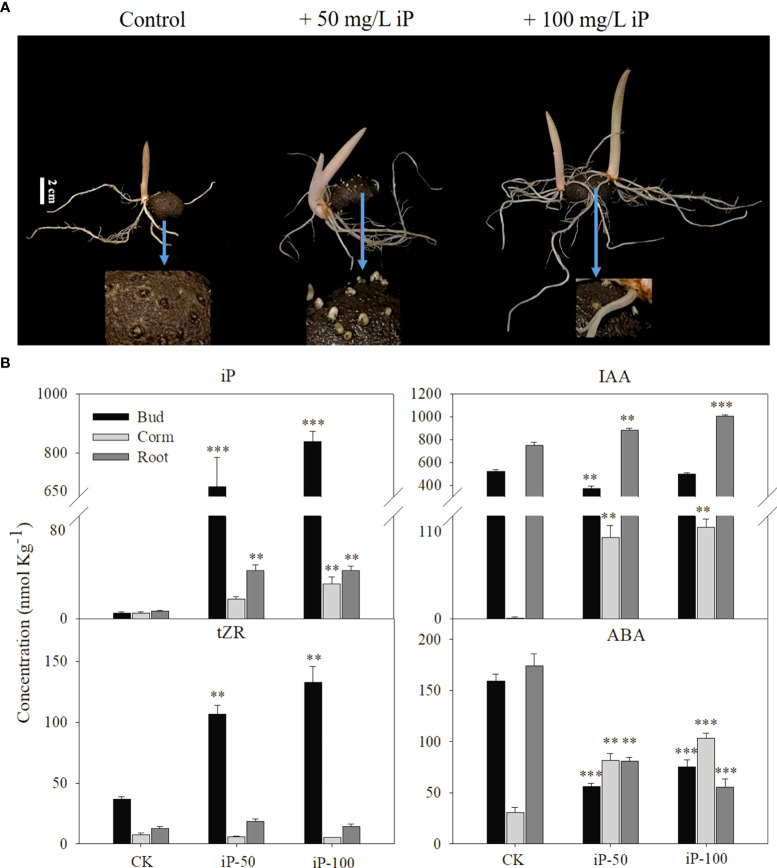
Effect of external application of iP on growth traits **(A)**: from leaf to right, dormant corms treated by water (control), 50 mg L^-1^iP and 100 mg L^-1^ iP and after 30 days. White bars = 2 cm. The content of iP, tZR, IAA and ABA contents of different tissues under iP treatment **(B)** Error bars represent the SE (n=3), and asterisks indicate significant differences compared with the control. *: *P* < 0.05; **: *P* < 0.01; ***: *P* < 0.001 (based on ANOVA, followed by Tukey’s post-hoc tests for comparison).

The content of hormones in roots, stems and buds at the sprouting stage was measured and the results showed that the iP levels in the buds, corms and roots were significantly increased under iP treatment (**Figure 3A**). Among them, the iP content in the buds was 838.5 nmol kg^-1^, which was 30.79 and 22.46 times higher than that in corms and roots respectively, accounting for approximately 93% of the whole plant content. In addition, the tZR content in buds was significantly higher under the iP treatment, but the content in corms and roots was not significantly different from that in the control. It is worth mentioning that iP treatment increased the IAA content in corms and roots, but 50 mg L^-1^ of iP treatment had an obvious suppressive effect on the IAA content in buds. Interestingly, the effect of iP treatment on the ABA content in each tissue was also inconsistent. The iP treatment significantly reduced the ABA content in both buds and roots but considerably increased the ABA content in corms (**Figure 3B**).

### 3.3 Expression of key genes in the cytokinin biosynthesis and degradation pathway

**Figure 4:**
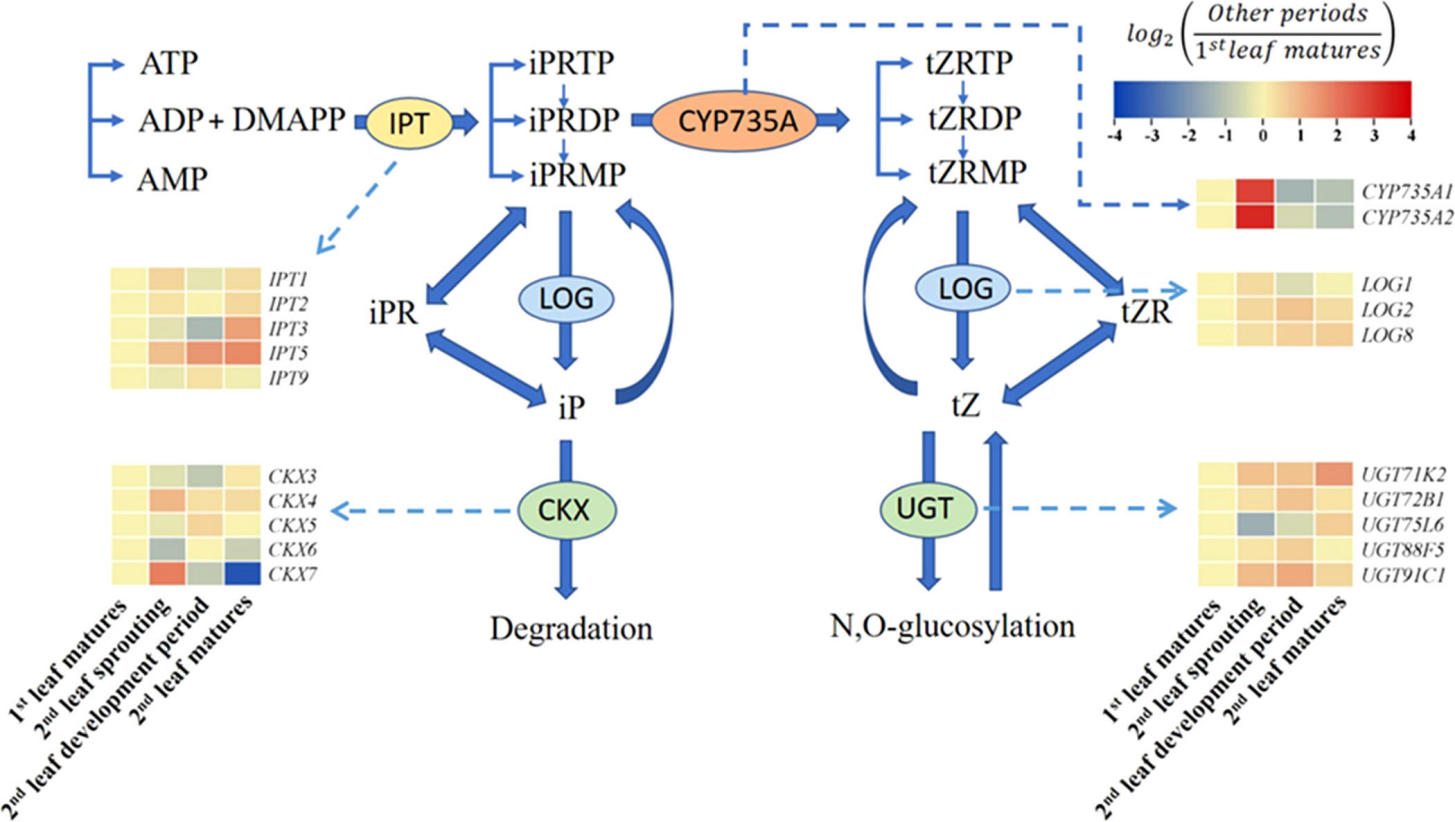
Summary of the cytokinin biosynthesis and catabolism pathways (modified from Osugi and Sakakibara, 2015). Changes in transcript levels are indicated by color codes. Red shows upregulated, and blue indicates downregulated of gene expression (using FPKM values) compared with first leaf matures period. Two color log scales are included.

To explore the transcriptional regulation of cytokinin biosynthesis across the growth cycle of *A. muelleri*, we examined the gene expression of *IPT* and *LOG* during the synthesis of IP or IPR and determined the expression of *CYP735A* during the synthesis of tZ or tZR (**Figure 4**). Compared to those in the first leaf matures period, the FC values of IPT3 and IPT5 in the second leaf matures period were 2.47 and 3.19, respectively. However, in the second leaf sprouting period, all IPTs detected in this study were not significantly changed compared to the nonsprouting stage. Instead, *CYP735A1* and *CYP735A2* tended to be highly expressed at the second leaf sprouting stage, and the expression increased 7.19- and 9.15-fold, respectively, compared to the non-sprouting stage. Regarding the expression of *LOGs*, all the *LOGs* increased at the second leaf sprouting stage compared to the nonsprouting stage, but none of the changes were significant. CKX and UGT are key enzymes in the degradation and transformation of iP and tZ, respectively. The expression of *CKX3*, *CKX5* and *CKX6* showed a decreasing trend with the sprouting of the second leaf, and *UGT75L6* also showed a decreasing trend in the same period.

### 3.4 Changes in IAA and ABA during the growth cycle of *Amorphophallus muelleri*

**Figure 5:**
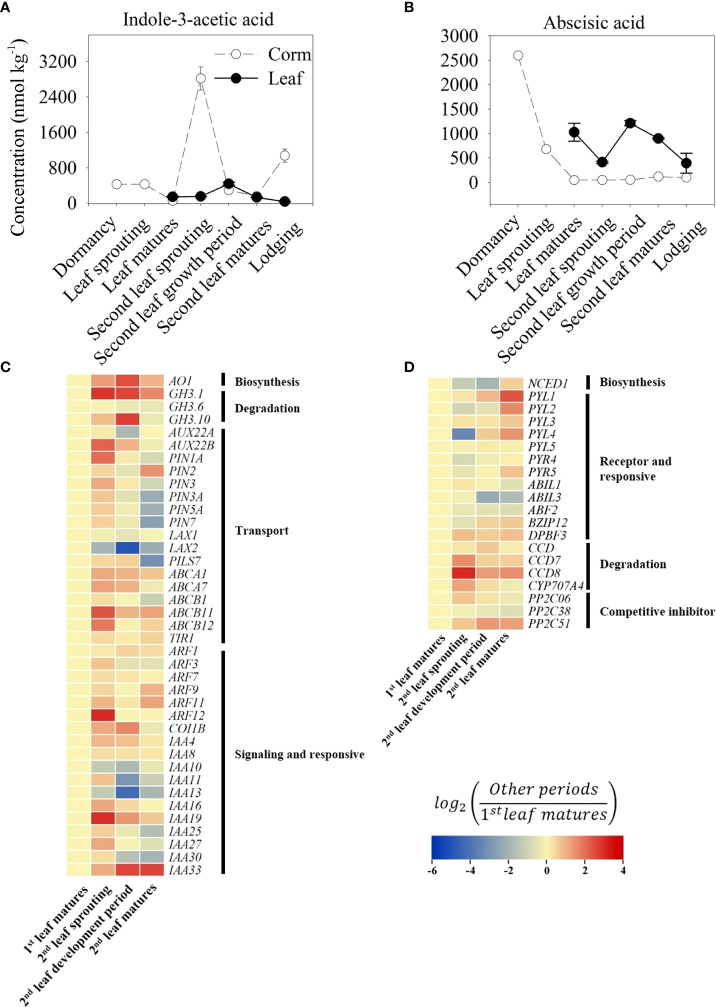
Trends of indole-3-acetic acid (IAA, **A**) and abscisic acid (ABA, **B**) content in growth cycle of *A. muelleri*. Error bars represent the SE (n=3), and different letters indicate significant differences compared with different growth periods (P<0.05, based on ANOVA, followed by Tukey’s post-hoc tests for comparison). Expression profile of transcripts involved in the IAA **(C)** and ABA **(D)** signaling pathways. Changes in transcript levels are indicated by color codes. Red shows upregulated, and blue indicates downregulated of gene expression (using FPKM values) compared with first leaf matures period. Two color log scales are included.

The content of IAA in corms was significantly higher at the second leaf sprouting stage, reaching a peak of 2816.94 nmol kg^-1^, which was 6.59 times higher than the content at the dormant stage in corms and 10.83 times higher than the content in the leaves (260.08 nmol kg^-1^, which was also the peak content in the leaves) during the same period (**Figure 5A**). Consistent with the high content of IAA at the second leaf sprouting stage, the genes related to IAA biosynthesis and transport, such as indole-3-acetaldehyde oxidase (AO1) and auxin efflux carrier PIN1A, presented relatively high expression levels in the same period (**Figure 5C**).

From the changes in ABA, the highest ABA content was found in the dormant corms, and with the appearance of leaf buds, the ABA content decreased considerably to only 26% of the dormant content and dropped to 46.84 nmol kg^-1^ at the first leaf maturation stage, which was less than 2% of that in the dormant period (**Figure 5B**). Similar to the trend for the ABA content, the expression of *NCED1*, a rate-limiting enzyme gene in ABA biosynthesis, was also suppressed at the second leaf sprouting stage. In contrast, genes associated with ABA degradation, such as carotenoid cleavage dioxygenase (CCD), or competitive inhibitors of ABA receptors, such as protein phosphatase (PP2C51), showed an increase in expression during the second leaf sprouting period (**Figure 5D**).

## 4 Discussion

### 4.1 High tZR content in the corms may be the key factor promoting second leaf sprouting

Konjac can form multiple dormant axillary buds on its corms; however, only one bud is activated and gives rise to a leaf in most konjac species throughout the growth cycle. Previous research has made great progress in enabling an understanding of the mechanisms of this apical dominance (Bainbridge et al., 2005; Dun et al., 2006; Teper-Bamnolker et al., 2012; Shi and Vernoux, 2022). However, the mechanism of breaking apical dominance for multileaf growth of pearl-bud-type konjac is not clear. The relative balance of auxin and cytokinins may be a central regulator of multileaf growth (Umehara et al., 2008; Fang et al., 2020).

In this study, the patterns of changes in the cytokinin contents in the corms across the growth cycle showed two different trends. One of the trends was represented by tZR, which was significantly higher at the second leaf sprouting stage. Surprisingly, while the expression of *CYP735A1* and *CYP735A2*, which synthesize the precursors for tZR (Takei et al., 2004; Osugi and Sakakibara, 2015), was highly elevated at the second leaf sprouting stage, the expression of *IPTs*, which have key roles in cytokinin biosynthesis, did not change significantly. In addition, most cytokinin contents in leaves during the same period were significantly lower than those in corms. Together with the fact that many cytokinins tend to be synthesized in the root (Nordström et al., 2004; Tanaka et al., 2006; Domagalska and Leyser, 2011), we speculate that the high cytokinin contents in the corms may come from the roots rather than being synthesized *de novo* in corms. The corms serve as the loci for bud germination. tZR is the most dominant cytokinin type in the corms, and its dramatically increased content may be closely related to the germination and growth of the second seedling. This indicates that the high tZR content in the corms may be the key factor promoting second leaf sprouting in *A. muelleri*.

Another trend was observed for iP-type cytokinins, which had the highest content in the dormant period and decreased with sprouting of the first leaf. iP-type cytokinins are the main form in phloem saps, and communicate acropetal and systemic long-distance signals (Hirose et al., 2008). The corm is the organ that connects the root to the leaf and acts as a reservoir organ, possibly retaining iP-type cytokinins during transport. The length of buds and roots from dormant corms treated with iP were significantly higher than those of the control, and the percentage of multibud plants increased under treatment. This implies that iP-type cytokinins tend to play a role in promoting first seedling sprouting.

### 4.2 The interactions of cytokinins, IAA and ABA are tissue specific

**Figure 6:**
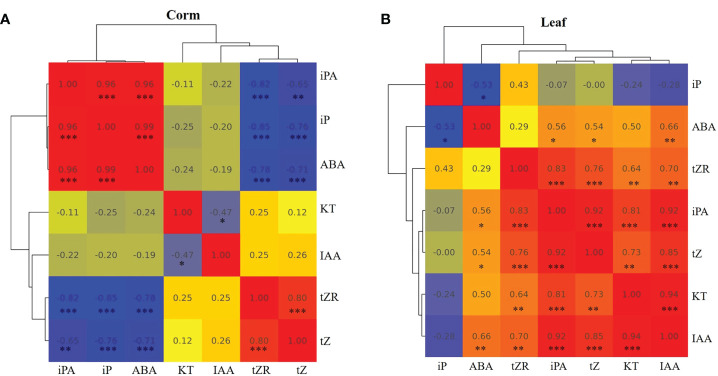
Pearson correlation analysis among the hormones in corms **(A)** and leaves **(B)** of *A. muelleri*. Red indicates a positive correlation, and blue indicates a negative correlation. The numbers represent the correlation coefficients. Asterisks denote significant levels, *: *P* < 0.05; **: *P* < 0.01; ***: *P* < 0.001.

Some studies have suggested that biologically active cytokinins can accumulate in dormant bulbs and may be involved in the process of breaking dormancy (Suttle, 2009; Hartmann et al., 2011). In our results, there was a high accumulation of iP-type cytokinins in dormant corms. The correlation analysis between hormones in corms and leaves also showed that iP and ABA were significantly positively correlated in corms (**Figure 6A**) but negatively correlated in leaves (**Figure 6B**). The external application experiment further demonstrated that iP treatment increased ABA content in corms while decreasing the content in roots and buds. This implies that different organs have different strategies for responding to iP.

It is generally accepted that cytokinins can promote bud outgrowth, but polar IAA transport forms the signal that inhibits lateral branching (Domagalska and Leyser, 2011). In our results, both the IAA and tZR contents in corms increased significantly during the second leaf sprouting period. Moreover, the genes encoding the family of auxin efflux carriers, such as *PINs*, tent to be highly expressed during the same period, which would facilitate efficient and directional auxin export out of the cell and promote the polar auxin transport stream, precluding upward movement into the axillary buds (Forestan and Varotto, 2012; Huang et al., 2020). This may allow cytokinins in corms to directly promote bud outgrowth. In addition, *COI1B*, which encodes the F-BOX protein in the IAA signaling pathway, was highly expressed during the second leaf sprouting period, and it could regulate the biosynthesis of cytokinins by repressing the expression of *IPTs* (Schaller et al., 2015; Kolachevskaya et al., 2021). This may also be an important reason why the expression of IPTs did not increase significantly during the second leaf sprouting period. This result also supports our speculation that the corm is not the main site of cytokinin biosynthesis.

In addition to the negative regulation of cytokinins by IAA, there are also some indications that the levels of cytokinins and IAA are positively correlated in some tissues (Li and Bangerth, 2003; Jones et al., 2010; Kolachevskaya et al., 2021). In our results, the IAA content in both roots and corms was significantly increased under iP treatment, which is consistent with the results of previous studies. However, we also found that iP treatment had an obvious inhibitory effect on the IAA content in the buds. This result indicates that there is tissue specificity in the interaction between cytokinins and IAA.

The control of bud outgrowth has been studied by many generations of researchers, and many models and hypotheses have been proposed to explain the mechanisms. This suggests that there is a more complex hormonal regulatory network to control lateral bud growth. In this study, we mainly focused on cytokinins and discussed the interaction between cytokinins and IAA and ABA during axillary bud growth. We found high levels of both tZR and IAA in the corms of the second leaf emergence stage, but there was tissue specificity in the interactions between cytokinin and IAA and ABA. Other hormones that are potentially involved in lateral bud germination, such as strigolactone and gibberellin, have not been studied in this study. In summary, our results provide a spatiotemporal variation pattern of hormones of the pearl-bud-type konjac, providing insights into the transcriptional control of major hormones involved in second bud germination. This work contributes to the understanding of the role of hormones in the multileaf growth type of konjac.

## Data availability statement

The original contributions presented in the study are publicly available. This data can be found here: NCBI, PRJNA863734. The raw data for the hormone measurements in this study can be found in https://osf.io/nsqf6/, DOI 10.17605/OSF.IO/NSOF6. The RNA-seq data used in this study are available in the Gene Expression Omnibus (NCBI) under code PRJNA863734.

## Author contributions

ZX performed most of the experiments and wrote the manuscript. YQ and LY conceived this study and prepared the initial outline. FH, JL, YK, HW and PG contributed to the statistical analysis and figure prepare. All authors contributed to the article and approved the submitted version.

## Funding

This research was financed by Yunnan Provincial Science and Technology Department (No. 2018HB100, 2019FH001-008, 2019FH001-051) and Project of China National Tobacco Corporation Yunnan Branch (No. 2021530000242017).

## Conflict of interest

The authors declare that the research was conducted in the absence of any commercial or financial relationships that could be construed as a potential conflict of interest.

## Publisher’s note

All claims expressed in this article are solely those of the authors and do not necessarily represent those of their affiliated organizations, or those of the publisher, the editors and the reviewers. Any product that may be evaluated in this article, or claim that may be made by its manufacturer, is not guaranteed or endorsed by the publisher.

## Supplementary material

Supplemental Table 1 Information about standards and stable isotope-labeled internal standards.

Supplemental Table 2 The concentration and RIN value of RNA extraction.

Supplemental Table 3 The growth traits of *A. muelleri* under iP treatment.

Supplemental Figure 1 The MS spectra figures of standard and sample solution.

Supplemental Figure 2 The quality of RNA extraction.
