# Supplementary figures and images for: A high trans-zeatin nucleoside concentration in corms may promote the multileaf growth of Amorphophallus muelleri
Source: Front Plant Sci. 2022 Oct 6;13:964003. doi: 10.3389/fpls.2022.964003 (PMC9583388; doi:10.3389/fpls.2022.964003)

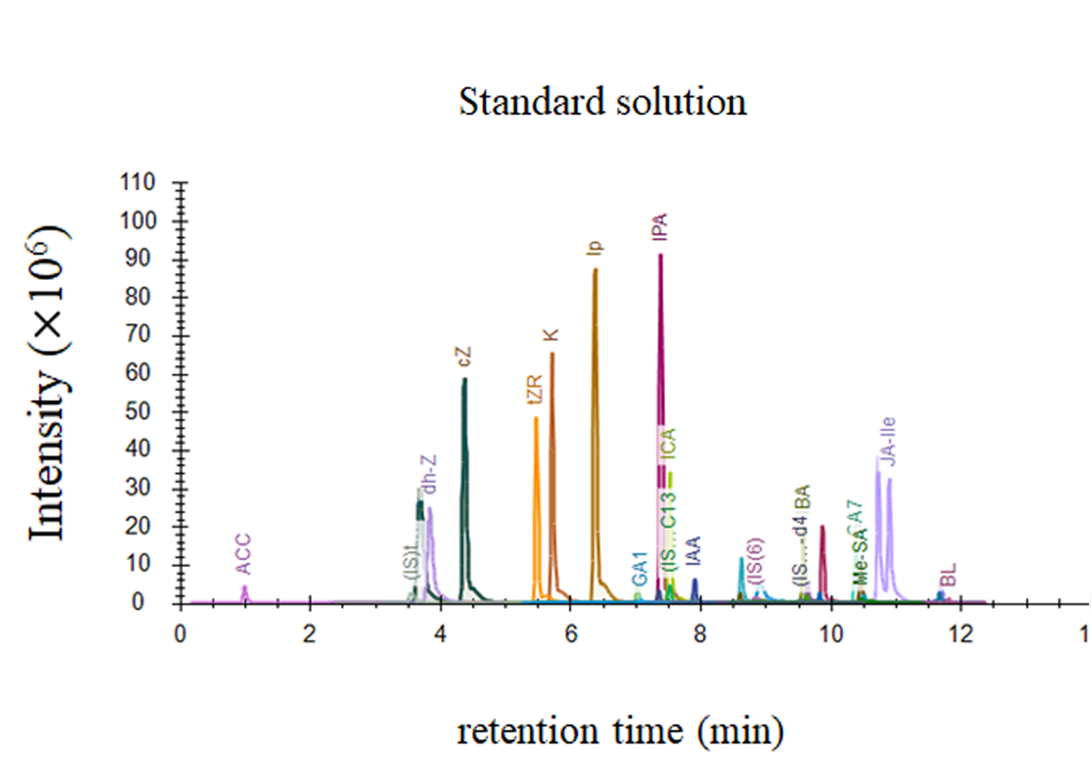

Supplement: Supplemental Figure 1 — The MS spectra figures of standard and sample solution. [file Image_1.tif]

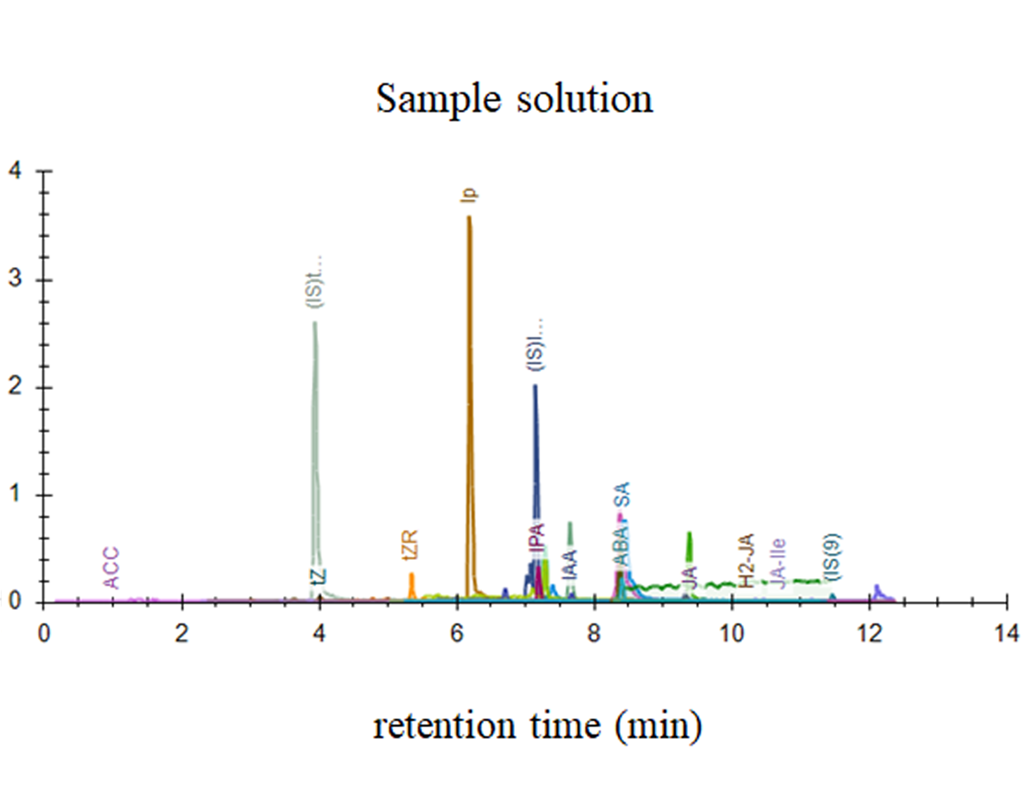

Supplement: Supplemental Figure 2 — The quality of RNA extraction. [file Image_2.tif]

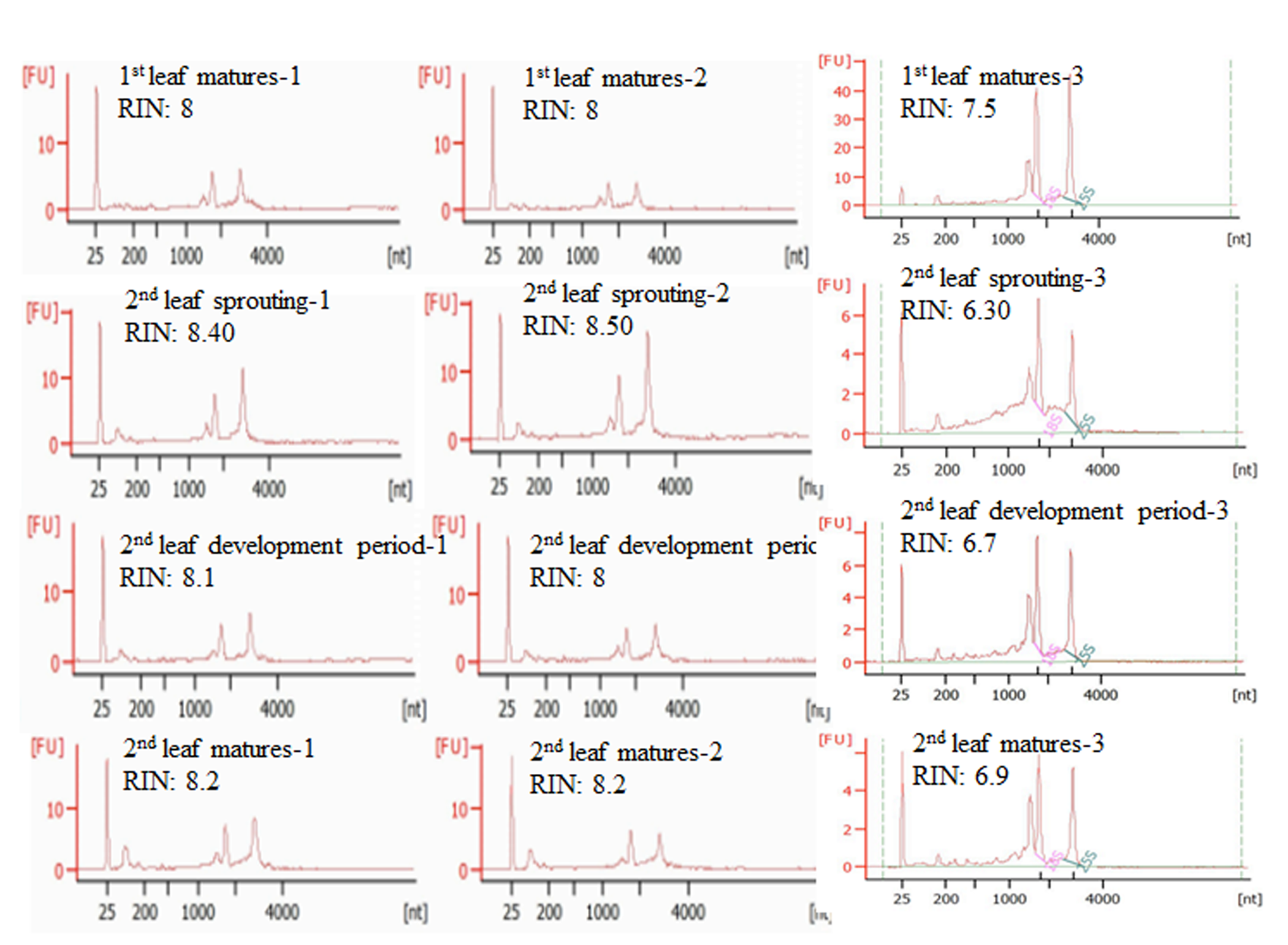

Supplement: Supplementary file 6 [file Image_3.tif]

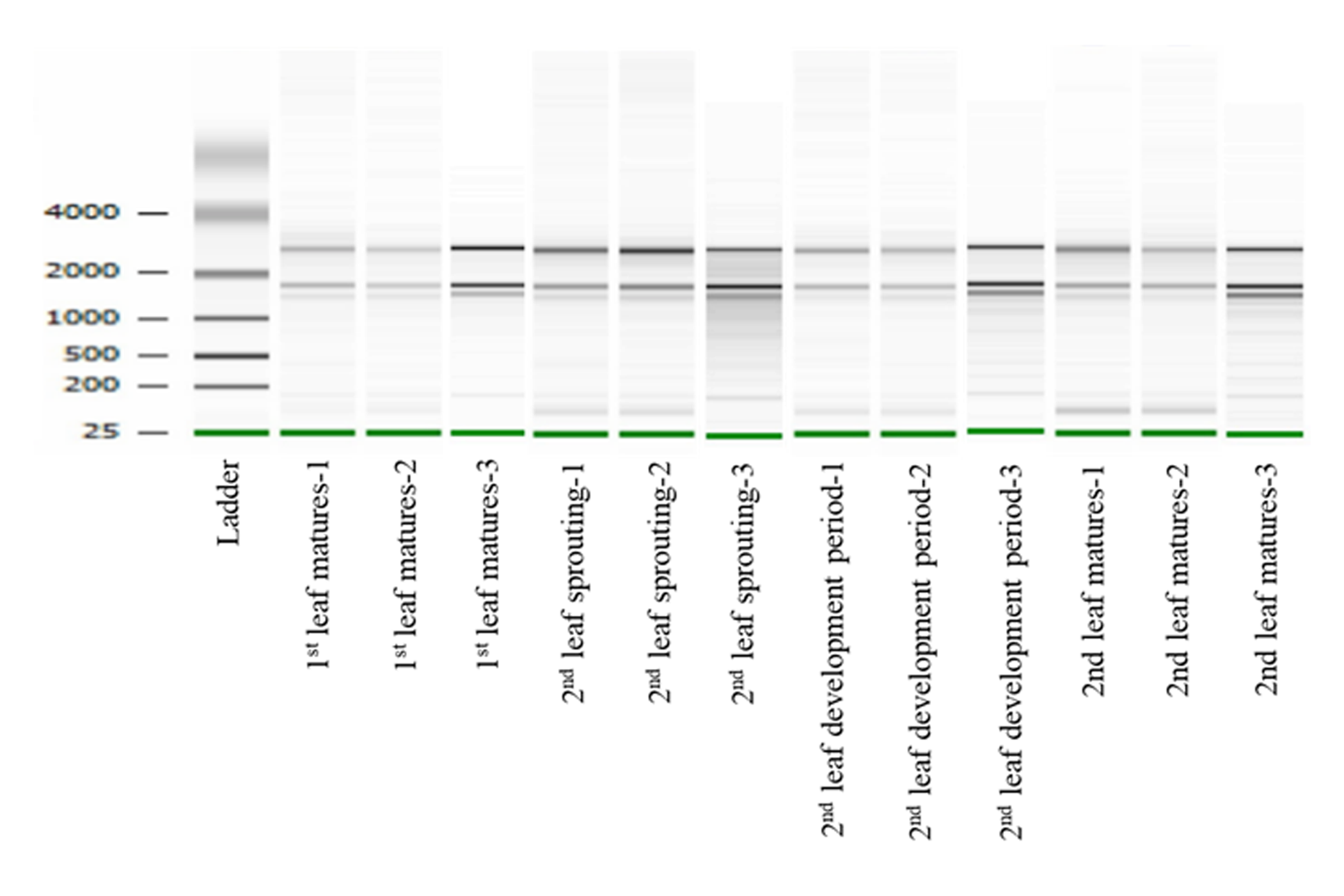

Supplement: Supplementary file 7 [file Image_4.tif]
